# Bioinformatic mapping of AlkB homology domains in viruses

**DOI:** 10.1186/1471-2164-6-1

**Published:** 2005-01-03

**Authors:** Marit S Bratlie, Finn Drabløs

**Affiliations:** 1 Department of Cancer Research and Molecular Medicine, Faculty of Medicine, MTFS, Norwegian University of Science and Technology, N-7489 Trondheim, Norway

## Abstract

**Background:**

AlkB-like proteins are members of the 2-oxoglutarate- and Fe(II)-dependent oxygenase superfamily. In *Escherichia coli *the protein protects RNA and DNA against damage from methylating agents. 1-methyladenine and 3-methylcytosine are repaired by oxidative demethylation and direct reversal of the methylated base back to its unmethylated form. Genes for AlkB homologues are widespread in nature, and Eukaryotes often have several genes coding for AlkB-like proteins. Similar domains have also been observed in certain plant viruses. The function of the viral domain is unknown, but it has been suggested that it may be involved in protecting the virus against the post-transcriptional gene silencing (PTGS) system found in plants. We wanted to do a phylogenomic mapping of viral AlkB-like domains as a basis for analysing functional aspects of these domains, because this could have some relevance for understanding possible alternative roles of AlkB homologues e.g. in Eukaryotes.

**Results:**

Profile-based searches of protein sequence libraries showed that AlkB-like domains are found in at least 22 different single-stranded RNA positive-strand plant viruses, but mainly in a subgroup of the *Flexiviridae *family. Sequence analysis indicated that the AlkB domains probably are functionally conserved, and that they most likely have been integrated relatively recently into several viral genomes at geographically distinct locations. This pattern seems to be more consistent with increased environmental pressure, e.g. from methylating pesticides, than with interaction with the PTGS system.

**Conclusions:**

The AlkB domain found in viral genomes is most likely a conventional DNA/RNA repair domain that protects the viral RNA genome against methylating compounds from the environment.

## Background

The purpose of this study has been to identify domains with homology to AlkB in viral genomes, in order to get a better understanding of distribution and possible function of such domains. The AlkB protein of *E. coli*, and probably most of its homologues, is involved in repair of alkylation damage in DNA and RNA. It repairs 1-methyladenine and 3-methylcytosine by oxidative demethylation and direct reversal of the methylated base back to its unmethylated form. Recently the protein was identified as a member of the 2-oxoglutarate (2OG)- and Fe(II)-dependent oxygenase superfamily [1-3]. The catalytic reaction requires molecular oxygen, Fe^2+ ^and 2-oxoglutarate, which is subsequently converted into succinate, CO_2 _and formaldehyde [4].

The 2OG-FeII oxygenase superfamily is widespread in Eukaryotes and bacteria [1], and is currently the largest known family of oxidising enzymes without a heme group [5]. The 3D structure of several of these oxygenases is known, and they share a common fold with a structurally conserved jelly roll β-sheet core with flanking α-helices. Very few residues are totally conserved across these structures, basically just the residues involved in coordination of the Fe(II) ion and the 2-oxoglutarate.

AlkB-like genes are widespread in most types of organisms except Archaea. However, whereas bacteria normally have just one or at most two AlkB homologues [6], multicellular Eukaryotes tend to have several homologues. In the human genome at least 8 different AlkB homologues (ABHs) have been identified [7]. These homologues seem to have slightly different properties with respect to substrate preference and subcellular localisation, and this may be a reason for the proliferation of ABHs e.g. in humans. However, a detailed functional mapping of all ABHs has not yet been carried out.

A sequence alignment of known ABHs identifies very few residues as totally conserved, basically just a HxD motif, a H and a RxxxxxR motif. These residues are also conserved in the more general 2OG-FeII oxygenase superfamily as described above, except for the final R. The first three residues (HxD and H) are involved in Fe(II)-coordination, whereas the first R is involved in 2OG-coordination. The final R is most likely involved in AlkB-specific substrate binding.

In addition to DNA repair, it has been shown that *E. coli *AlkB and the human AlkB homologue hABH3 may be involved in RNA repair. When expressed in *E. coli *both AlkB and hABH3 reactivate methylated RNA bacteriophage MS2 *in vivo*. This illustrates that direct repair may be an important mechanism for maintenance of RNA in living cells [4]. RNA repair proceeds by the same mechanism as DNA repair. Repair of damaged RNA was previously considered very unlikely, due to the natural redundancy of RNAs in a cell [8]. However, RNA is essential for cell function: unrepaired RNA can lead to miscoded or truncated proteins, and alkylated RNA could signal cell cycle checkpointing or apoptosis [9]. Consequently the occurrence of RNA repair does not come as a great surprise. The mechanism of direct reversal of methylation as used by AlkB homologues is particularly important for RNA repair, as it means that single-stranded regions may be repaired without introducing strand breaks. Repair of alkylation damage in DNA and RNA has recently been reviewed [10].

AlkB homologues have also been found in plant viruses. It has been suggested that methylation may be used in host-mediated inactivation of viral RNAs, and that AlkB homologues in some plant viruses may be used to counteract such defence mechanisms [1]. However, no detailed study of this has been published.

The research project reported here has focused on a better understanding of the distribution and potential function of putative AlkB homology domains by using *in silico *mapping of viruses in which such domains have been found, as well as related viruses.

## Results

The general mapping strategy of the project was to identify viral genomes with AlkB homology domains, identify common features of these genomes, and subsequently find additional genomes with similar features, but without AlkB homology domains. This data set could then be used to analyse the properties and distribution of AlkB-like domains in viruses, as a basis for generating hypotheses about the evolution and function of these domains.

### Identification of relevant viral protein sequences

The PSI-Blast search for viruses in the NCBI nr protein sequence database was initiated with ALKB_ECOLI (NCBI gi113638), restricted to residues 110 to 210 and using the default inclusion threshold of 0.005 on E-values. The chosen residue range corresponds to the most conserved region in AlkB homologues [10].

The PSI-Blast search converged after 4 iterations, and included 43 hits below the 0.005 inclusion threshold, from 22 different ssRNA positive-strand viruses. The AlkB homologues were found in viruses belonging to *Allexi*, *Ampelo*, *Carla*, *Fovea*, *Mandari*, *Potex*, *Tricho *and *Vitiviruses*, all of which are known to infect plants [11].

**Figure 1:**
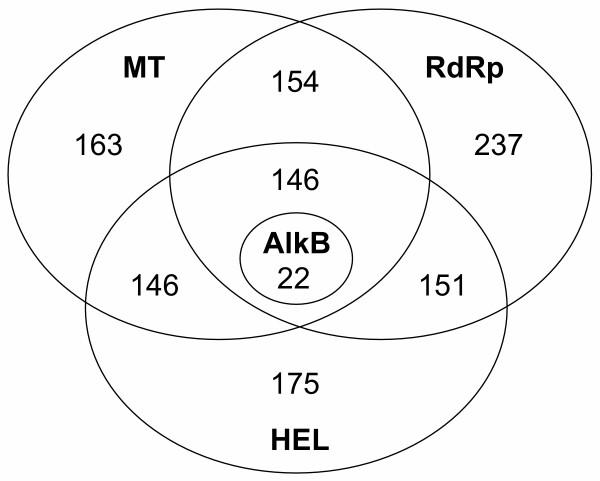
PSI-Blast search results shown as a Venn diagram. Initial searches using methyltransferase, helicase and RdRp domains retrieved 163, 175 and 237 sequences, respectively. A total of 146 sequences contained all three domains, and 22 of these also contained an AlkB domain.

In all of these viruses the AlkB domain is a part of the replicase polyprotein, which normally consists of a viral methyltransferase domain (MT), a viral helicase domain (HEL) and a RNA-dependent RNA polymerase domain (RdRp). Therefore separate PSI-Blast searches for the individual components of the replicase polyprotein were also initiated. All searches were done with PSI-Blast using the default inclusion threshold (E-value of 0.005). The searches for MT and HEL domains were initiated using residue ranges 449–841 and 1938–2178 respectively from *Grapevine leafroll-associated virus 3 *(*Ampelovirus*, NCBI gi29650261). The search for RdRp was initiated with residue range 1361–1798 from *Soil-borne cereal mosaic virus *(*Furovirus*, NCBI gi11546056). These sequences were chosen based on the output from the previous AlkB search. This gave a library of protein sequences with either AlkB, MT, HEL or RdRp domains, the general composition of which is illustrated in Figure 1. From this library a subset was generated, consisting of all sequences containing MT, HEL and RdRp domains. This included processed (cleaved) polyprotein sequences where RdRp was found as a separate subsequence. However, whenever possible, the protein sequence corresponding to the genomic sequence was used. The final library, described in Table 1 and in Additional file 1, consisted of 146 sequences from a large number of different viruses.

**Table 1:** Summary of Pfam domains

Classification				Pfam domains^b^				
Host	Family	Genus	n^a^	AB	OT	PC	A1	ot

**Plant**	*Bromoviridae*	*Alfamovirus*	1					
		*Bromovirus*	4					4
		*Cucumovirus*	3					
		*Ilarvirus*	11					
		*Oleavirus*	1					
		Unassigned	1					
	
	*Closteroviridae*	*Ampelovirus*	4	2				
		*Closterovirus*	5			1		
		*Crinivirus*	4					
	
	*Flexiviridae *1	*Allexivirus*	5	1				
		*Mandarivirus*	1	1				
		*Potexvirus*	17	3				
	
	*Flexiviridae *2	*Capillovirus*	3			3		3
		*Carlavirus*	6	5	5	6		
		*Foveavirus*	6	5	6	6		
		*Trichovirus*	2	2		2		
		*Vitivirus*	2	2				
		Unassigned	2	1	2	2		
	
	*Tymoviridae*	*Maculavirus*	1			1		
		*Marafivirus*	3			3		3
		*Tymovirus*	7			7		3
	
	Unassigned	*Benyvirus*	2			2		
		*Furovirus*	4					
		*Hordeivirus*	1					
		*Idaeovirus*	1					
		*Pecluvirus*	1					
		*Pomovirus*	4					
		*Tobamovirus*	18					
		*Tobravirus*	3					
		Unassigned	2					

**Invertebrate**	*Tetraviridae*	*Betatetravirus*	1					
		Unassigned	1					

**Vertebrate**	*Togaviridae*	*Alphavirus*	17				17	2
	
	*Unassigend*	*Hepatitis E-like*	2				2	

^a ^Number of sequences.

^b ^Number of sequences with each domain type, excluding the common MT, HEL and RdRp domains (AB – AlkB, OT – OTU, PC – Peptidase C, A1 – A1pp, ot – other).

The library of protein sequences was screened for known domains in Pfam. This identified Pfam domains *Viral_helicase1 *and *RNA_dep_RNApol2 *in all sequences, corresponding to HEL and RdRp domains, respectively. In addition *Vmethyltransf *and *2OG-FeII_Oxy*, corresponding to MT and 2OG-FeII oxygenase (AlkB) domains, were identified in several sequences. However, for sequences from *Flexiviridae *and *Tymoviridae *there was no clear identification of any MT domain by Pfam, although they had been retrieved by PSI-Blast in a search for MT domains. Therefore HMMER was used to build a Pfam type profile for these sequences. A PSI-Blast search was initiated using residues 1–500 of *Potato virus M *(*Carlavirus*, NCBI gi9626090). Twelve representative sequences were selected from the search output, covering *Carla*, *Fovea*, *Potex*, *Allexi*, *Capillo *and *Maculavirus*. Subsequences representing the conserved region according to the PSI-Blast alignment, corresponding to residues 35–378 of the query sequence, were aligned using ClustalX, and a Pfam type profile was generated and calibrated using tools from the HMMER package. The resulting profile was able to identify putative methyltransferase domains in all *Flexiviridae *and *Tymoviridae *sequences in the data set.

Other Pfam domains – *Peptidase_C21*, *C23*, *C33*, *C34*, *C35 *and *C41*, *A1pp *and *OTU *– were also identified in subsets of sequences. *A1pp *is a member of the Appr-1-p processing enzyme family, and the domain is found in a number of otherwise unrelated proteins, including non-structural proteins of several types of ssRNA viruses. *OTU *is a member of a family of cysteine proteases that are homologous to the ovarian tumour (*otu*) gene in *Drosophila*. Members of this family are found in Eukaryotes, viruses and pathogenic bacteria.

### Phylogenetic analysis

**Figure 2:**
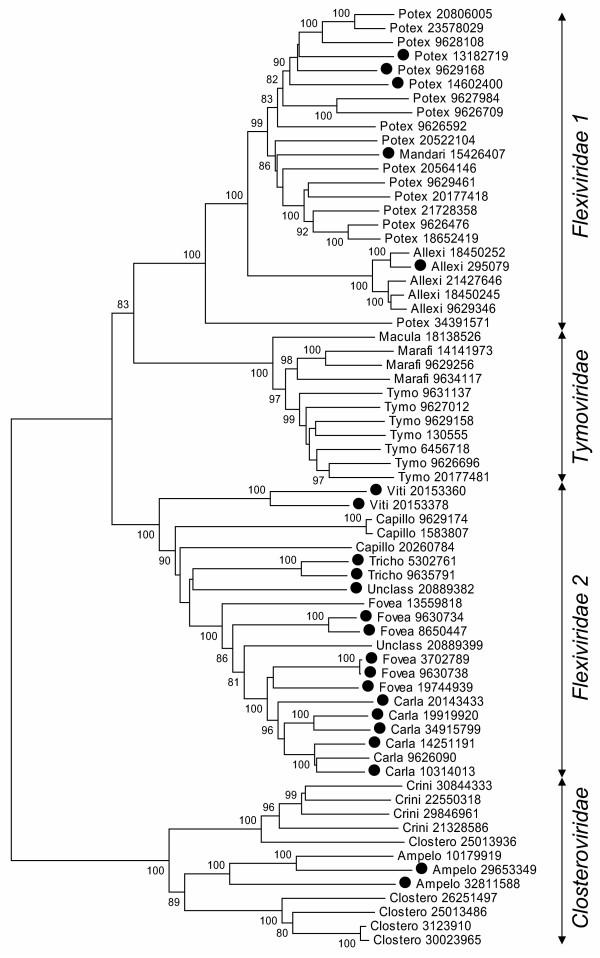
Unrooted phylogenetic tree for *Flexiviridae *1 and 2, *Tymoviridae *and *Closteroviridae*. Sequences are labelled with genus and NCBI gi accession number. Bootstrap values ≥ 80 are shown. Sequences with AlkB domains are indicated with black dots.

The MT, HEL and RdRp domains identified by Pfam as described above were extracted from the library sequences, aligned by ClustalX, and combined into a new alignment consisting of only these domain regions. This turned out to be necessary in order to get robust alignments. The intervening regions between the conserved domains are extremely variable in these sequences, and this tended to confuse alignment programs in the sense that conserved regions were not correctly aligned. The combined sequence alignment of domains from *Closteroviridae*, *Flexiviridae *and *Tymoviridae *was then used as input for building a phylogenetic tree with MEGA2. The final tree is shown in Figure 2, with polyproteins containing AlkB-like domains indicated.

A second alignment was generated from all sequences with AlkB-like domains, using only the regions corresponding to MT, AlkB, HEL and RdRp Pfam domains. The domains were aligned individually, and the combined alignment was used as input for MEGA2. However, this data set did not give a reliable phylogeny (data not shown), and the separate domains of this alignment were therefore analysed individually and compared. This analysis is summarised in Table 2. For each domain a bootstrapped neighbour-joining (NJ) tree was generated with MEGA2. The average bootstrap support value over all branches was computed for each tree, and this value was clearly lower for the AlkB tree compared to the other trees. A maximum likelihood (ML) tree was generated for each domain with Tree-Puzzle. This showed the same trend, the likelihood values indicated that the AlkB tree was clearly inferior to the other trees. The individual trees were then compared using the quartet-based strict joint assertions (SJA) measure as implemented in the Component software package. Both the NJ and ML trees showed the same trend. The MT, HEL and RdRp domains gave similar tree structures, with SJA values between 0.053 and 0.161 for NJ trees and between 0.058 and 0.092 for ML trees when they were compared to each other. The AlkB domain gave a significantly different tree structure, with SJA values from 0.456 to 0.524 for NJ trees and from 0.258 to 0.317 for ML trees when compared to the MT, HEL and RdRp trees (the actual trees are given in Additional file 2). For comparison the SJA values for comparing the corresponding NJ and ML trees for MT, AlkB, HEL and RdRp were 0.054, 0.000, 0.040 and 0.003, respectively, showing that the NJ and ML procedures gave almost identical tree structures. Day has estimated expectation values and standard deviations for various distance measures (including SJA) for comparison of random trees [12]. The SJA values shown in Table 2 for comparisons between MT, HEL and RdRp NJ trees were 14.2 – 17.1 standard deviations from the expectation value of 0.665 for a tree with 22 nodes, whereas the corresponding values for the AlkB NJ tree were 4.4 – 5.4 standard deviations from the expectation value. Similar ranges were observed for the ML trees as well as for alternative distance measures, e.g. the Symmetric Difference (SD) measure (data not shown). Although this means that the SJA value for comparing AlkB trees to MT, HEL and RdRp trees were significantly better than for random trees, it also shows that the MT, HEL and RdRp trees were clearly more similar to each other than to the AlkB tree.

**Table 2:** Strict joint assertions distances for NJ and ML trees

ML\NJ^a^	**MT**	**AlkB**	**HEL**	**RdRp**	**log L**^b^	**BS (%)**^c^	**ID (%)**^d^
**MT**	-	0.488	0.161	0.053	-14068	85	27
**AlkB**	0.263	-	0.524	0.456	-4016	35	38
**HEL**	0.058	0.317	-	0.117	-10425	87	28
**RdRp**	0.062	0.258	0.092	-	-14543	91	37

^a ^Strict joint assertions (SJA) values based on quartets as computed by Component for ML trees (lower left) and NJ trees (upper right). SJA is defined as resolved and different quartets divided by all resolved quartets.

^b ^The likelihood value from Tree-Puzzle.

^c ^Average bootstrap value for all branches in each NJ tree.

^d ^Average sequence identity for all pairs of sequences in each alignment.

**Figure 3:**
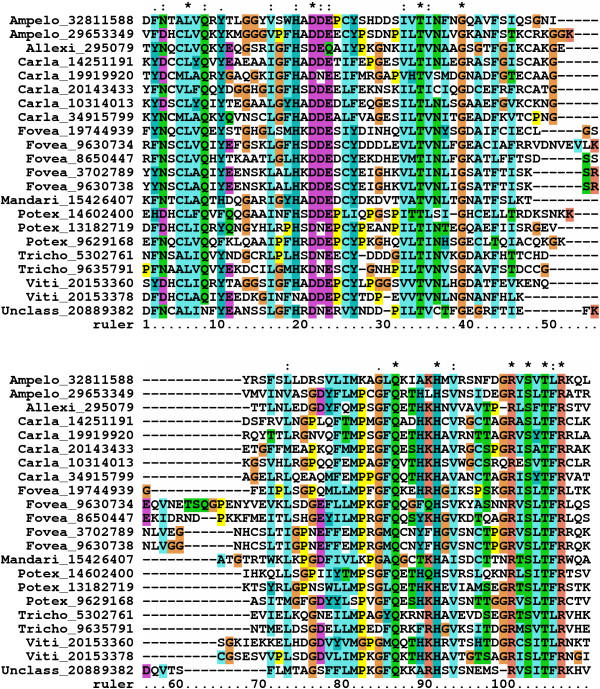
Multiple alignment of sequence regions corresponding to the AlkB domains. The alignment was generated with ClustalX. The residues involved in coordination of the essential Fe^2+ ^ion are completely conserved, except in one of the *Vitivirus *sequences. These residues are the HxD motif, a single H, and the first R in the RxxxxxR motif. The function of the remaining conserved residues is unclear, but at least some of them may be involved in coordination of the substrate [10].

The alignment of the AlkB domain seemed to be of comparable quality to the other alignments. In fact the AlkB domain had the highest average pairwise sequence identity, as seen in Table 2 (see Figure 3 for the actual alignment). In other words, these AlkB domains were as similar to each other as the other three domains with respect to sequence identity, but they did not represent a consistent evolutionary history when compared to the other domains of this polyprotein. This may indicate that the AlkB domains have evolved separately from the other domains, and possibly as several independent instances.

**Figure 4:**
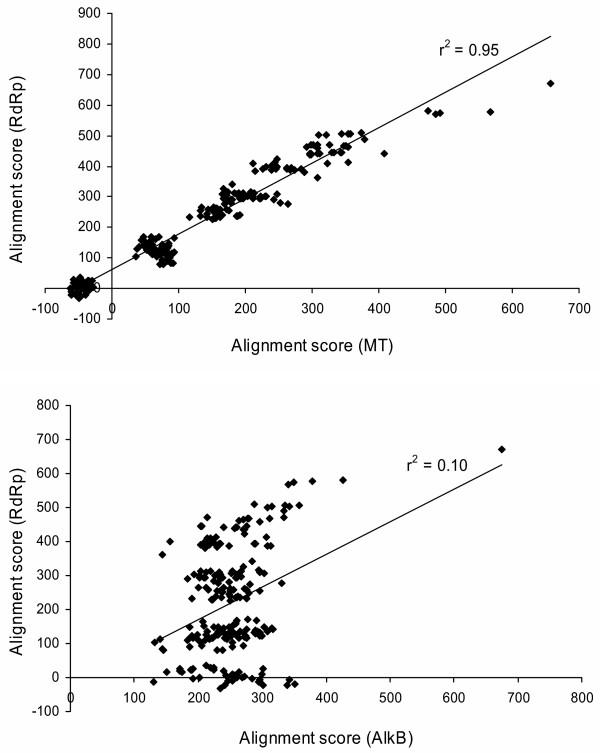
Pairwise distances between sequence regions corresponding to methyltransferase (MT), RdRp and AlkB domains. Each data point corresponds to e.g. RP-RP and MT-MT distances for the same pair of sequences, and sequences showing similar evolutionary distance in these two regions will fall on the diagonal. The pairwise distances were estimated from multiple alignments using the Blosum50 score matrix [47]. Trend lines were estimated with Excel. The trend line for AlkB vs. RdRp is heavily influenced by the point at (675, 670). It represents two *Foveavirus *sequences (NCBI gi3702789 and gi9630738), they are 98% identical over the full polyprotein sequence.

The degree of co-evolution was analysed by computing pairwise distances between sequence regions in the alignment of MT, AlkB, HEL and RdRp domains described above. In Figure 4 selected results are shown as scatter plots, where the Blosum 50 score value between e.g. the MT domains in a pair of sequences is plotted against the score value for AlkB domains in the same pair of sequences. Plots for the MT, HEL and RdRp domains show that they are strongly correlated for MT vs. RdRp (r^2 ^= 0.95), MT vs. HEL (r^2 ^= 0.87) and HEL vs. RdRp (r^2 ^= 0.81). The plot of the AlkB domain vs. these three domains for the same set of sequences shows a very low degree of correlation for AlkB vs. RdRp (r^2 ^= 0.10), AlkB vs. MT (r^2 ^= 0.12) and AlkB vs. HEL (r^2 ^= 0.16).

**Figure 5:**
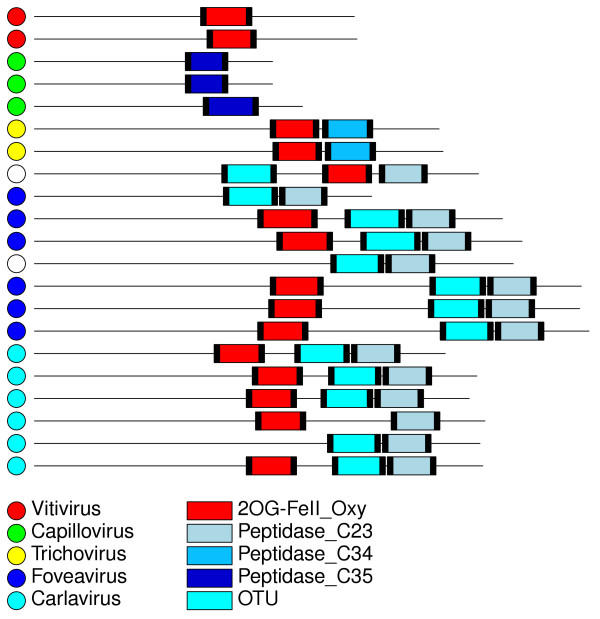
Location of Pfam domains in the variable region of *Flexiviridae *2 sequences. The regions have been extracted directly from Pfam output, and sequences and regions are drawn to scale. The black bar at each end of a motif indicates that a full-length motif has been found, for partial motifs the bar at the truncated end would be missing.

As mentioned above the genome organisation of these replicase polyprotein sequences seems to be very flexible. In order to analyse domain organisation the location of identified Pfam domains were plotted for a number of sequences, as shown in Figure 5.

### Similarity of viral AlkB domains to other AlkB sequences

The results described above may indicate that the AlkB domains have been integrated into the replicase polyprotein relatively recently (see Discussion). In order to test for potential sources selected AlkB domains were compared to non-viral sequences. PSI-Blast was used to search the NCBI nr database, removing all viral hits in the final search report. Most of the remaining top-scoring hits were from bacteria. This included two different strains of *Xanthomonas, X. axonopodis *pv *citri *and *X. campestris *pv *campestris*. *Xanthomonas *attacks plants such as citrus, beans, grapevine, rice and cotton [13]. The search also returned high-scoring hits from another plant pathogen, *Xylella fastidiosa*. This bacterium infects a great variety of plants, including grapevine, citrus, periwinkle, almond, oleander and coffee [14].

### Potential similarities in variable regions

Pfam searches obviously will only identify known domain types in protein sequences. In order to identify potential similarities in regions that were not recognised by Pfam, systematic PSI-Blast searches were performed, using the polyprotein regions between the MT and HEL domains and searching against the NCBI database of reference sequences [15], excluding all viral entries. A maximum of 5 PSI-Blast iterations were allowed, with an inclusion threshold of 0.005. The expected homologues of the AlkB-domain were found with high confidence, as most of the E-values were < 1 × 10^-50^. Homologues of typical viral domains like the viral peptidases were obviously not found, as all viral database entries were excluded. Very few new similarities were found by these searches. *Pepper ringspot virus *(*Tobravirus*, NCBI gi20178599) showed significant similarity to site-specific DNA-methyltransferase from *Nostoc *sp (E = 1 × 10^-74^), as well as other cytosine 5C-specific DNA methylases. *Bamboo mosaic virus *(*Potexvirus*, NCBI gi9627984) showed similarity to aggregation substance Asa1 from *Enterococcus faecalis *(E = 6 × 10^-34^). A small number of additional similarities seemed to be caused by biased sequence properties (e.g. proline-rich regions), and were probably not significant. This included matches against mucin and cadherin-like proteins from *Homo sapiens *and multidomain presynaptic cytomatrix protein (piccolo) from *Rattus norvegicus*. In general the variable regions seemed to be truly variable, with very little similarity to other proteins, except for the Pfam domains already identified.

### Loss of domains in related polyprotein sequences

**Figure 6:**
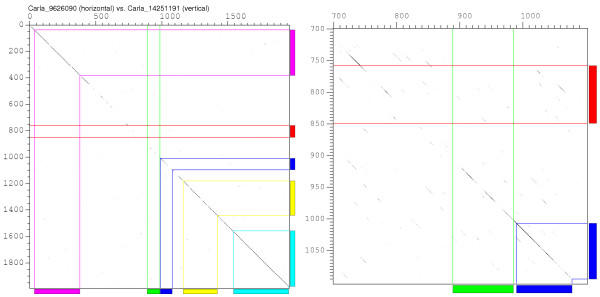
Dot plots for *Potato virus M *(NCBI gi9626090) and *Aconitum latent virus *(NCBI gi14251191). To the left the full sequences are shown, using the program default for similarity threshold, and to the right the region with AlkB, OTU and peptidase integration, using a slightly lower (more sensitive) threshold for sequence similarity. The Pfam regions corresponding to MT (magenta), AlkB (red), OTU (green), peptidase (blue), HEL (yellow) and RdRp (cyan) domains are indicated.

As seen in Figures 2 and 5, some closely related sequences are lacking specific domains in the sense that HMMER does not find a significant similarity to the Pfam entries for these domains. In order to understand the degree of sequence variation associated with this domain loss, as well as the general sequence variation in conserved vs. non-conserved regions of typical polyproteins, several dot plots were generated. The dot plot for two *Carlavirus *sequences, *Potato virus M *(NCBI gi9626090) and *Aconitum latent virus *(NCBI gi14251191), is shown in Figure 6. The dot plot confirms that these two sequences are closely related in the MT, HEL and RdRp domains. However, there are significant differences in the region between MT and HEL. *Potato virus M *is lacking the AlkB domain whereas *Aconitum latent virus *is lacking the OTU domain. As seen from the dot plot, short regions of similarity close to the diagonal shows that both domains may have been present in an ancestral sequence. However, this region shows a high degree of sequence variation, and as indicated by the dot plot they are almost exclusively mutations. Non-essential or non-functional domains are probably rapidly lost. In this particular case, none of the typical AlkB motifs seem to be conserved in *Potato virus M*, indicating that this indeed is a non-functional AlkB domain.

## Discussion

### The N-terminal domains of *Flexiviridae *and *Tymoviridae *are methyltransferases

As described above the Pfam methyltransferase motif (*Vmethyltransf*) did not match any of the putative methyltransferase domains of *Flexiviridae *and *Tymoviridae*, despite the fact that they had been identified via PSI-Blast searches starting with known methyltransferases. Therefore an additional Pfam-type profile was generated. It is obviously a possibility that these domains in *Flexiviridae *and *Tymoviridae *are not methyltransferases, and that they are false positives from PSI-Blast. However, the essential residues of a typical viral methyltransferase motif are conserved in the alignment of these domains (data not shown) [16]. In *Bamboo mosaic virus*, which belongs to *Flexiviridae*, the residues H68, D122, R125 and Y213 have been identified as putative active site residues with similarity to the Sindbis virus-like methyltransferase [17], and it has been demonstrated that this region of the *Bamboo mosaic virus *has methyltransferase activity, as it catalyses the transfer of a methyl group from S-adenosylmethionine (AdoMet) to GTP or guanylylimidodiphosphate (GIDP). The corresponding sequence positions are almost completely conserved in the alignment of *Flexiviridae *and *Tymoviridae *N-terminal domains. This is most likely significant, as only 7 positions in total are completely conserved in this alignment, which means that the majority of the conserved positions are known to be essential for methyltransferase activity. Work e.g. by Hataya *et al*. seems to support the assumption that this sequence region is a methyltransferase domain [18]. It therefore seems likely that all the sequences with AlkB domains also contain functional MT, HEL and RdRp domains. The MT domains are probably involved in capping of genomic and subgenomic RNA [19].

### The viral AlkB domains are most likely functional

Based on the bioinformatic evidence generated here, it seems reasonable to assume that the viral AlkB domains identified by Pfam are functional. All the essential residues found in 2-oxoglutarate- and Fe(II)-dependent oxygenases are conserved, in particular the putative Fe^2+ ^coordinating H, D and H residues at alignment positions 19, 21 and 91 of Figure 3, and the 2-oxoglutarate coordinating R at position 100. The conserved R at position 106 is also very characteristic of AlkB homologues [10]. The fact that all AlkB-like domains identified in these viral genomes are full-length, compared to the Pfam profile, also seems to support the hypothesis that these domains are functional.

### The AlkB domains are found in a subset of viral genomes

The Pfam searches show that AlkB domains are found only in a subset of the viral genomes. This subset is phylogenetically consistent (see Figure 2), as it is mainly restricted to the *Flexiviridae*, and in particular to a subset of the *Flexiviridae *consisting of *Viti*, *Capillo*, *Tricho*, *Fovea *and *Carlavirus*. This subset is well separated from the remaining *Flexiviridae *in the phylogenetic analysis. The split seems to be robust from bootstrap analysis, therefore this family will be discussed here as two subfamilies, *Flexiviridae *1 and 2. The same split was observed by Adams *et al*. in their recent analysis of the *Flexiviridae *family [20]. Most of the AlkB domains (15) are found in *Flexiviridae *2. The remaining AlkB domains are found in *Flexiviridae *1 (5) and *Closteroviridae *(2). In general, all the *Flexiviridae *2 sequences have at least one extra domain in addition to MT, HEL and RdRp: either AlkB, OTU-like cysteine protease or a peptidase. Most other plant viruses that are included in this survey do not have additional domains, except for *Tymoviridae *where a peptidase domain seems to be common. For the remaining plant virus families included here (excluding *Tymoviridae *and *Flexiviridae *2), only 14% seem to have additional domains.

### Introduction of AlkB domain in plant virus is probably a recent event

The observed distribution of AlkB domains could most easily be explained by assuming that an ancestral AlkB domain was integrated into the genome of the last common ancestor of the *Flexiviridae *2 subfamily. Subsequent virus generations derived from this common ancestor would then also contain an AlkB domain, except in those cases where the domain was lost again. This scenario could also include subsequent transfer to a small number of other virus families e.g. by recombination.

If this scenario was correct, then one would expect the different domains of the polyprotein to have a similar evolutionary history. From the phylogenetic analysis (Table 2) this seems to be confirmed for the MT, HEL and RdRp domains, but not for the AlkB domain. This indicates that the AlkB domain may not have co-evolved with the other domains, at least until relatively recently. This seems to be confirmed by looking at the degree of co-evolution, which was analysed by computing pairwise distances between alignment regions representing the relevant domains (Figure 4). In the case of perfect co-evolution all points should fall on a diagonal. This seems to be the case for the MT, HEL and RdRp domains. However, the plot of the AlkB domain vs. these three domains for the same set of sequences does not show a similar correlation. Only some of the closely related sequence pairs in the upper right quadrant of the plot in Figure 4 show some degree of correlation for AlkB vs. RdRp. The most likely explanation seems to be that most of the AlkB domains have not co-evolved with the other domains for any significant period of time. This seems to rule out the possibility of ancient integration of the AlkB domain, except if we assume that an ancient viral AlkB domain has frequently recombined with other AlkB domains. However, it is difficult to distinguish a scenario with frequent recombination of AlkB domains from *de novo *integration, and the net effect on the properties observed here would be the same.

As seen in Figure 4, the range of score values is generally smaller for the AlkB domains than e.g. the RdRp domains, particularly if we exclude a couple of very high-scoring cases (see figure caption). On the other hand, the degree of sequence variation within the collection of AlkB domains is significant, average sequence identity for pairwise alignments is 38%, and only 10% of the positions are totally conserved. This can be consistent with a recent integration if we assume that several different AlkB-type vectors have been used for integration (see below for details). An increased mutation rate after integration could also have contributed to sequence diversity in this region. Moving the AlkB domain into a novel structural and functional context would have removed many of the original evolutionarily constraints, as well as introduced some new ones. This could have created a "punctuated equilibrium" type of situation, potentially leading to a very rapid evolution that could have introduced significant differences between the AlkB domains, independent of the evolution in the other domains. A high mutation rate seems to be the case for this region in general, as indicated in Figure 6. Although the MT, HEL and RdRp domains seem to be well conserved from the dot plot, there are very large sequence variations in the intervening region. One sequence in Figure 6 has a well conserved AlkB domain, the other an OTU domain. The fact that there are very weak sequence similarities in these two domains in the dot plot indicates that both sequences originally had both domains. However, the fact that this similarity now is very weak and without any of the typical AlkB active site motifs also indicates a high mutation rate where non-essential domains are rapidly lost. Therefore the conservation of AlkB domains is a strong indication that they are functional, as already mentioned.

### The AlkB domains may represent several separate integrations

If we assume that AlkB domains have been integrated relatively recently, then either *de novo *integration or recombination (horizontal gene transfer) may have been the main driving force for spreading the AlkB domain to new genomes. In the first case a large number of individual integrations could have lead to the present situation. If horizontal gene transfer was the main driving force, the initial number of integrations might have been quite small. It is not easy to differentiate between these two situations.

The map of Pfam motifs in the variable region between the MT and HEL domains in *Flexiviridae *2 polyproteins (Figure 5) shows that they have a very similar domain organisation, basically an AlkB domain followed by an OTU domain and a peptidase domain, located towards the C-terminal part of the sub-sequence. The relatively constant domain organisation seems to be consistent with a small number of initial integrations that were subsequently diffused to related genomes e.g. by homologous recombination. However, this is not fully consistent with the fact that the viruses with AlkB domains have been collected from hosts at very different locations, e.g. Canada, USA, Russia, Italy, Germany, France, India, Taiwan, China and Japan. Although import of virus-infected species or transmission by insects may transport viruses over significant distances, it is not obvious that this is enough to explain the observed distribution of AlkB-like domains. Therefore several independent integrations, mainly from closely related hosts, have to be considered as an alternative explanation. This explanation seems to be supported by the apparent lack of any consistent evolutionary relationships between the various AlkB domains, as seen in Table 2. It is not easy to see how this model can be consistent with the observed similarities in domain organisation in *Flexiviridae*. Assuming that this region has a high degree of variability, one would expect the variability to affect localisation of integrated domains as well. However, it is possible that conserved regions e.g. in the polyprotein play a significant role in integration of novel domains. It may be relevant in this context that preliminary simulations indicate that e.g. the AlkB domains tend to form independent folding domains in the folded RNA structure of the polyprotein RNA (F. Drabløs, unpublished data). This property may possibly facilitate the insertion of such domains into the viral genome.

### The original AlkB integration may be of bacterial origin

There are many groups of organisms that can act as vectors and spread viruses, including bacteria, fungi, nematodes, arthropods and arachnids. The plant viruses may have acquired the AlkB domain either from the vector or from the host itself. As already mentioned, searching with viral AlkB domains in protein sequence databases resulted mainly in bacterial sequences, including the plant pathogens *X. fastidiosa *and *campestris*. It is therefore a reasonable possibility that AlkB domains in plant viruses have originated from bacterial mRNA. It is also possible that the mRNA originated from other vectors or from the host itself, but at the present time this is not easily verified or disproved because of the limited number of insect and plant genomes that have been sequenced.

### The AlkB domain probably protects virus RNA against methylation

It has previously been suggested that the viral AlkB domain may be involved in protecting the virus against the post-transcriptional gene silencing (PTGS) system of the host [1]. PTGS is known as one of a plant's intrinsic defence mechanisms against viruses [21]. Gene silencing can occur either through repression of transcription (transcriptional gene silencing – TGS) or through mRNA degradation, PTGS. The PTGS-mechanism in plants shows similarities to RNA interference (RNAi) in animals [22]. This mechanism results in the specific degradation of RNA. Degradation can be activated by introduction of transgenes, RNA viruses or DNA sequences homologous to expressed genes [23]. Many viruses have developed mechanisms to counteract PTGS in order to successfully infect plants [24]. Two of these suppressors of PTGS have been identified as Hc-Protease and the 2b protein of *Cucumber mosaic virus *[25]. Although both proteins suppress PTGS, it is likely that they do so via different mechanisms. Could the AlkB-like domain found in some of the plant viruses also be a suppressor of PTGS? Previously reported research indicates that methylation of transcribed sequences is somehow connected with PTGS, and the methylation can be mediated by a direct RNA-DNA interaction [26]. This RNA-directed DNA methylation has been described in plants, and leads to *de novo *methylation of nearly all cytosine residues within the region of sequence identity between RNA and DNA [27]. Both RNA methylation and methylation of host proteins that are essential for viral replication would be detrimental to the virus. It has already been mentioned that AlkB repairs 1-methyladenine and 3-methylcytosine by oxidative demethylation. It is therefore possible that AlkB demethylates the nucleotides methylated by the PTGS mechanism, helping the virus to overcome one of the major defence mechanisms of the plant.

As shown here, only a subset of plant viruses have the AlkB domain. However, other viruses may be utilising naturally occurring AlkB proteins in the host. Viruses have to rely on a number of host proteins in order to replicate [28]. In some cases it is probably beneficial for the virus to integrate such genes into their own genome in order to ensure that they are accessible, although there will be a trade off between this advantage and the increased cost of maintaining a larger genome [29].

However, there is an alternative hypothesis with respect to the AlkB integration that also has to be considered. As discussed above, the AlkB domain seems to have been integrated relatively recently in viruses found at very different geographical locations, and the only obvious connection seems to be that most viruses belong to a subset of the *Flexiviridae*. However, the source of these viruses points at another common feature. As seen from the table given in Additional file 1, AlkB domains are often found in viruses associated with grapevine, apple, cherry, citrus and blueberry – crops where the usage of pesticides is common. It is known that several common pesticides (e.g. methyl bromide and some organophosphorus compounds) may cause methylation of DNA and RNA [30-33]. An integrated repair domain for methylation damage as part of the viral replication complex would therefore give the virus a competitive advantage in a highly methylating environment. The application of such pesticides would probably also stimulate AlkB production e.g. in co-infecting bacteria, giving these viruses easy access to AlkB mRNA for integration into their RNA genome.

It could be argued that a more active PTGS system in these plants would give a similar effect. However, in that case we would expect to see more ancient integrations of AlkB domains. It could also be argued that the presence of AlkB domains may be an artefact caused by promiscuous viral domains picking up available mRNA sequences during cultivation of viruses in the laboratory. However, given the large number of different laboratories involved, and the number of different hosts used (data not shown), this seems to be a very unlikely explanation.

The hypothesis that environmental compounds, in particular pesticides, may have provoked the integration of AlkB domains into the viral genomes depends upon a high mutation rate and frequent integrations of non-viral domains. The integrations have to be recent, not only in relative terms, compared to other domains in the same genome, but also in absolute terms, compared to the progress of modern agriculture. The integrations also have to be frequent, in the sense that it is likely that integration could have happened several times, in different biotopes.

It is difficult to estimate mutation rates in RNA viruses. They evolve very rapidly, and it is often difficult to assign reliable phylogenies. However, recent studies indicate that most ssRNA viruses have a mutation rate close to 10^-3 ^substitutions per site per year [34], e.g. the SARS virus has 1.16–3.30 × 10^-3 ^non-synonymous substitutions per site per year, which is considered to be a "moderate" ssRNA mutation rate [34]. If we assume that most ssRNA viruses have effective mutation rates within the same order of magnitude, a realistic mutation rate for the viruses included here might be something like 2.0 × 10^-3^. In that case, the MT, HEL and RdRp trees shown in Additional file 2 represent approximately between 325 and 750 years of evolution. In general the NJ trees estimate a slightly shorter evolutionary history (between 325 and 450 years) compared to the ML trees (between 550 and 750 years). In this estimate the *Ampelovirus *sequences have not been included, as they seem to have diverged from the remaining AlkB-containing viruses at a much earlier stage. If we believe that the AlkB integrations happened after the divergence of most sequence included here, as indicated by the lack of co-evolution in Figure 4, it does not seem unrealistic to assume that most of these integrations happened within the last 50 – 100 years or so. This estimate is of course very approximate, in particular since we do not know the true mutation rate of these viruses. However, it shows that a likely time span for AlkB integration is compatible with the evolution of modern agriculture. Unfortunately, because of the lack of any robust phylogeny for the viral AlkB sequences it does not make sense to do a similar estimate for that domain.

Although it is generally accepted that viruses frequently use recombination to acquire functionality [35], it is less well known how often this includes nonviral sequences. However, there are some well-documented examples, and in particular the properties of the ssRNA positive-strand *Pestivirus *may be relevant in this context. There are two biotopes of the pestiviruses, cytopathogenic (cp) and noncytopatogenic (noncp). The host is infected by the noncp form which is converted into the cp form by integration of a fragment of a cellular gene into the viral genome [36]. This introduces a protease cleavage site in the polyprotein. However, the important point here is that this happens as part of the normal infection process. It has been suggested that the integration is facilitated by the viral polymerase undergoing two subsequent template switches during minus-strand synthesis [37], although nonreplicative RNA recombination also may be a possibility [38]. Integration of cellular sequences have also been observed in other viruses, e.g. in influenza virus [39]. This shows that at least some viruses do have efficient mechanisms for recruitment of host genes into the viral genome. Therefore a recent and rapid integration of AlkB domains into selected plant virus genomes does not seem to be an unlikely scenario.

This study has focused on the AlkB domain, mainly as an attempt to get a better understanding of potential functions associated with this domain. However, it is likely that additional information about integration patterns and the relative importance of *de novo *integration vs. recombination can be achieved by a closer investigation of the other variable domains, e.g. by looking at how they correlate with the evolution of the AlkB domains.

## Conclusions

We believe that the viral AlkB-like domains are conventional repair domains targeted towards the viral RNA. The integration of AlkB domains into viral genomes may have been provoked by environmental methylating agents, e.g. the introduction of DNA/RNA-methylating pesticides in farming. The hypothesis [1] that the domain interferes with the PTGS system of plants can not be excluded, but seems to be less consistent with observed features of the AlkB integration.

## Methods

The NCBI nr protein sequence database was searched with PSI-Blast [40], with the output limited to viral sequences. Multiple alignments were made with ClustalX version 1.8 [41]. The phylogenetic tree in Figure 2 was made from ClustalX alignments by MEGA2 [42], using the neighbour-joining (NJ) approach with complete deletion of gap positions, Poisson correction of distances and 500 bootstrap steps. Phylogenetic trees for sequence regions from sequences with AlkB domains were made with the NJ approach as described above, but with 10.000 bootstrap steps. Corresponding trees were also made by the maximum likelihood approach (ML) by Tree-Puzzle version 5.2 [43], using an exact likelihood function, the VT matrix [44] and 10.000 puzzling steps. The trees from Tree-Puzzle were visualised with TreeView version 1.6.6 [45], and the NJ and ML trees were compared with Component version 2.0 [46]. Significance of pairwise tree distances were estimated using the data of Day [12]. Pairwise distances between sequences, for comparing evolution of AlkB domains to other viral domains, were computed directly from ClustalX alignments with local tools, using the Blosum50 mutation matrix [47], but without any correction for multiple substitutions. Motifs in protein sequences were identified using HMMER version 2.3.2 [48] with the Pfam library version 11.0 [49]. A Pfam-type profile for the methyltransferase domains of *Flexiviridae *and *Tymoviridae *was generated from a ClustalX alignment, using hmmbuild and hmmcalibrate from the HMMER package. Visualisation of motif positions in viral sequences was generated directly from the HMMER output files using a local tool as an interface to the GNU [50] groff software. Systematic large scale searches with polyprotein subsequences were done locally with PSI-Blast and the NCBI reference sequence database [15]. Dot plots for comparison of viral protein sequences were generated with Dotter version 3.0 [51].

## List of abbreviations used

MT – Methyl transferase; HEL – Helicase; RdRp – RNA-dependent RNA polymerase; ssRNA – Single-stranded RNA; PTGS – Post-transcriptional gene silencing; 2OG – 2-oxoglutarate; (h)ABH – (human) AlkB homologue; OTU – Ovarian tumour-like protein; NJ – Neighbour-joining; ML – Maximum likelihood; SJA – Strict joint assertions.

## Authors' contributions

MSB carried out all PSI-Blast searches, generated local (sub)sequence databases, and drafted the initial manuscript. FD conceived the study, carried out HMMER/Pfam searches, and estimated evolutionary distances. Both authors participated on sequence alignment, phylogenetic analysis and writing of the manuscript. Both authors have read and approved the final manuscript.

## Supplementary Material

Additional File 1 Full listing (with GI numbers) of viral sequences and domains included in the analysis. Click here for file

Additional File 2 Individual NJ and ML trees for relevant domains (MT, AlkB, HEL, RdRp). Click here for file
