# Supplementary material for: Bioinformatic mapping of AlkB homology domains in viruses
Source: BMC Genomics. 2005 Jan 3;6:1. doi: 10.1186/1471-2164-6-1 (PMC544882; doi:10.1186/1471-2164-6-1)
Supplement: Additional File 1 — Full listing (with GI numbers) of viral sequences and domains included in the analysis. [file 1471-2164-6-1-S1.pdf]

Additional file 1

Marit S. Bratlie and Finn Drabløs, “Bioinformatic mapping of AlkB homology domains in viruses”

Virus polyproteins identified by PSI-Blast searches for methyltransferase, viral helicase and RNA-dependent RNA polymerase domains. Domains verified using public Pfam profiles are shown in black, domains recognised only by local Pfam profiles or showing low similarity are shown in grey. MT – methyltransferase; AB – AlkB; OT – OTU-like; PC – Peptidase C; VH – viral helicase; A1 – Appr-1-p processing enzyme; RP – RNA-dependent RNA polymerase; ot – other. Please see the main paper for details.

**Classification**	**Virus**	**Accession**								
**Host** *Family* Genus	Species	NCBI gi-number	MT	AB	OT	PC	VH	A1	RP	ot
**Plant**										
Bromoviridae										
Alfamovirus	Alfalfa mosaic virus	75586, 75591	■				■		■	
Bromovirus	Broad bean mottle virus	21426915, 21426913	■				■		■	■
Bromovirus	Brome mosaic virus	9626932, 137271	■				■		■	■
Bromovirus	Cowpea chlorotic mottle virus	137252, 137272	■				■		■	■
Bromovirus	Spring beauty latent virus	22550378, 22531642	■				■		■	■
Cucumovirus	Cucumber mosaic virus	2313063, 9632337	■				■		■	
Cucumovirus	Peanut stunt virus	9632347, 9632349	■				■		■	
Cucumovirus	Peanut stunt virus (strain J)	75584, 137275	■				■		■	
Ilarvirus	American plum line pattern virus	13183015, 19697301	■				■		■	
Ilarvirus	Apple mosaic virus	19744911, 19744913	■				■		■	
Ilarvirus	Citrus leaf rugose virus	20087065, 20087063	■				■		■	
Ilarvirus	Elm mottle virus	20143445, 20143443	■				■		■	
Ilarvirus	Hydrangea mosaic virus	9622515, 9622510, 9622476	■				■		■	
Ilarvirus	Spinach latent virus	20522135, 20522137	■				■		■	
Ilarvirus	Tobacco streak virus	20564168, 20564165	■				■		■	
Ilarvirus	Tomato aspermy virus	20564161, 20564163	■				■		■	
Ilarvirus	Tulare apple mosaic virus	20564214, 12698358	■				■		■	
Ilarvirus	Prune dwarf virus	1762420, 14582299	■				■		■	

Ilarvirus	Prunus necrotic ringspot virus	24817631, 24817633
Oleavirus	Olive latent virus 2	1149529, 1149531
Unassigned	Pelargonium zonate spot virus	20177485, 20177487
Closteroviridae		
Ampelovirus	Little cherry virus 2	32811588, 32811589
Ampelovirus	Pineapple mealybug wilt-ass. virus 1	18253958, 18253959
Ampelovirus	Pineapple mealybug wilt-ass. virus 2	10179919, 10179920
Ampelovirus	Grapevine leafroll-associated virus 3	29653349
Closterovirus	Beet yellows virus	25013486
Closterovirus	Citrus tristeza virus	26251497
Closterovirus	Grapevine leafroll-associated virus 2	3123910, 3123911
Closterovirus	Grapevine rootstock stem lesion ass.	30023965
Closterovirus	Little cherry virus 1	25013936
Crinivirus	Cucumber yellows virus	29846961
Crinivirus	Cucurbit yellow stunting disorder v.	30844333
Crinivirus	Sweet potato chlorotic stunt virus	22550318
Crinivirus	Lettuce infectious yellows virus	21328586
Flexiviridae 1		
Allexivirus	Garlic virus A	18450252
Allexivirus	Garlic virus C	18450245
Allexivirus	Garlic virus E	21427646
Allexivirus	Garlic virus X	9629346
Allexivirus	Shallot virus X	295079
Mandarivirus	Indian citrus ringspot virus	15426407
Potexvirus	Bamboo mosaic virus	9627984
Potexvirus	Cactus virus X	14602400
Potexvirus	Cassava common mosaic virus	9628108
Potexvirus	Clover yellow mosaic virus	13182719
Potexvirus	Cymbidium mosaic virus	9629461
Potexvirus	Foxtail mosaic virus	9626709
Potexvirus	Narcissus mosaic virus	9626476

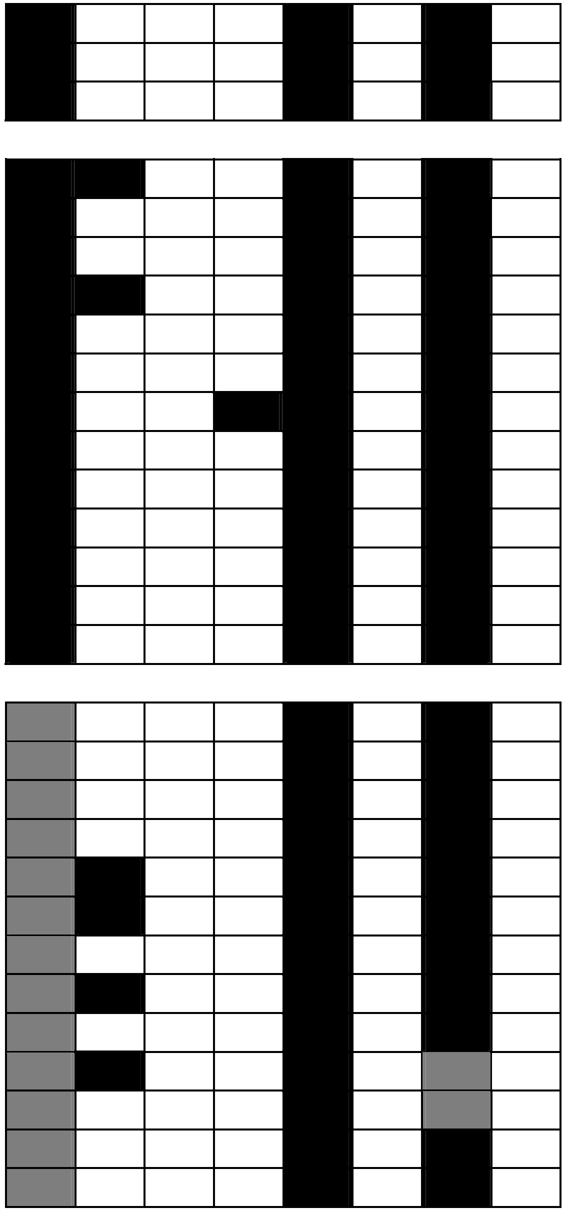

Potexvirus	Papaya mosaic virus	9629168
Potexvirus	Pepino mosaic virus	21728358
Potexvirus	Plantago asiatica mosaic virus	20806005
Potexvirus	Potato aucuba mosaic virus	20177418
Potexvirus	Potato virus X	9626592
Potexvirus	Scallion virus X	18652419
Potexvirus	Sclerotinia sclerotiorum deb. ass. v.	34391571
Potexvirus	Strawberry mild yellow edge virus	20522104
Potexvirus	Tulip virus X	23578029
Potexvirus	White clover mosaic virus	20564146
Flexiviridae 2		
Capillovirus	Apple stem grooving virus	9629174
Capillovirus	Cherry virus A	20260784
Capillovirus	Citrus tatter leaf virus	1583807
Carlavirus	Potato virus M	9626090
Carlavirus	Aconitum latent virus	14251191
Carlavirus	Blueberry scorch virus	19919920
Carlavirus	Garlic latent virus	20143433
Carlavirus	Hop latent virus	10314013
Carlavirus	Lily symptomless virus	34915799
Foveavirus	Apple stem pitting virus	19744939
Foveavirus	Banana mild mosaic virus	13559818
Foveavirus	Cherry green ring mottle virus	9630734
Foveavirus	Cherry necrotic rusty mottle virus	8650447
Foveavirus	Grapevine rupestris stem pitting ass.	3702789
Foveavirus	Rupestris stem pitting ass. virus-1	9630738
Trichovirus	Apple chlorotic leaf spot virus	5302761
Trichovirus	Cherry mottle leaf virus	9635791
Vitivirus	Grapevine virus A	20153360
Vitivirus	Grapevine virus B	20153378
Unassigned	Citrus leaf blotch virus	20889382

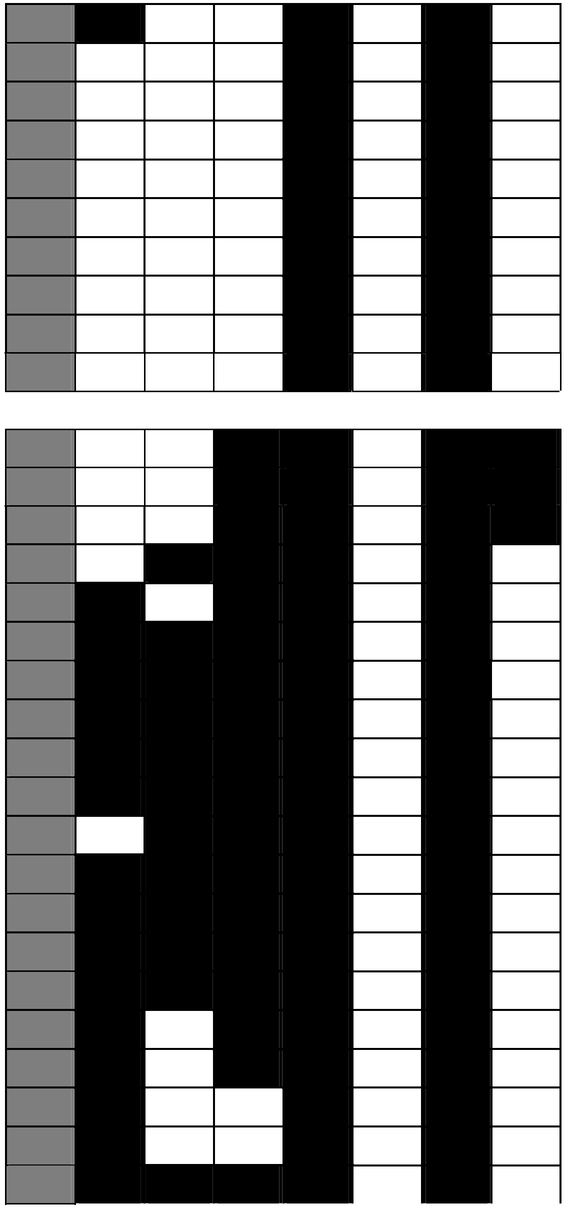

Unassigned	Sugarcane striate mosaic ass. virus	20889399
Tymoviridae		
Maculavirus	Grapevine fleck virus	18138526
Marafivirus	Maize rayado fino virus	14141973
Marafivirus	Oat blue dwarf virus	9629256
Marafivirus	Poinsettia mosaic virus	9634117
Tymovirus	Chayote mosaic tymovirus	6456718
Tymovirus	Eggplant mosaic virus	9626696
Tymovirus	Erysimum latent virus	9631137
Tymovirus	Kennedya yellow mosaic virus	9629158
Tymovirus	Ononis yellow mosaic virus	9627012
Tymovirus	Physalis mottle virus	20177481
Tymovirus	Turnip yellow mosaic virus	130555
Unassigned		
Benyvirus	Beet necrotic yellow vein virus	19919908
Benyvirus	Beet soil-borne mosaic virus	19919962
Furovirus	Chinese wheat mosaic virus	14270340
Furovirus	Soil-borne cereal mosaic virus	11546056
Furovirus	Soil-borne wheat mosaic virus	14330786
Furovirus	Sorghum chlorotic spot virus	21450885
Hordeivirus	Barley stripe mosaic virus	19744918, 19744920
Idaeovirus	Raspberry bushy dwarf virus	20386797
Pecluvirus	Peanut clump virus	20178622
Pomovirus	Beet soil-borne virus	19919936
Pomovirus	Beet virus Q	19919943
Pomovirus	Broad bean necrosis virus	25141248
Pomovirus	Potato mop-top virus	20376949
Tobamovirus	Chinese rape mosaic virus	7444535
Tobamovirus	Crucifer tobamovirus	18254497
Tobamovirus	Cucumber fruit mottle mosaic virus	12018228
Tobamovirus	Cucumber green mottle mosaic virus	19908652

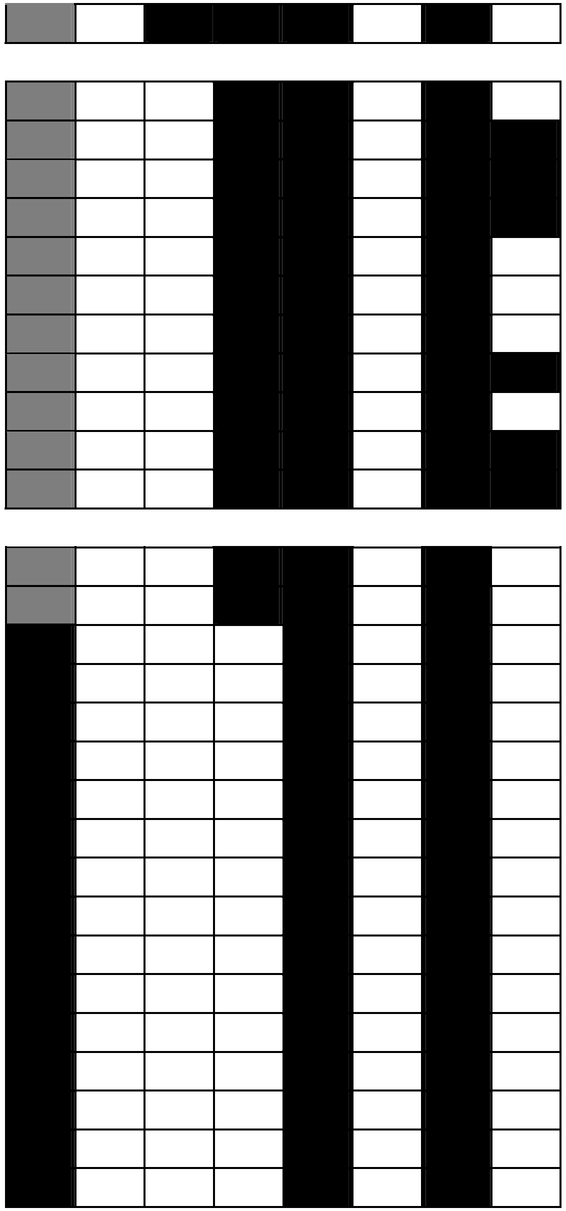

Tobamovirus	Kyuri green mottle mosaic virus	20153396
Tobamovirus	Obuda pepper virus	20806011
Tobamovirus	Odontoglossum ringspot virus	2339961
Tobamovirus	Paprika mild mottle virus	22212888
Tobamovirus	Pepper mild mottle virus	15387665
Tobamovirus	Ribgrass mosaic virus	14142012
Tobamovirus	Sunn-hemp mosaic virus	12643499
Tobamovirus	Tobacco mild green mosaic virus	18253267
Tobamovirus	Tobacco mosaic virus	1890235
Tobamovirus	Tomato mosaic virus	13357206
Tobamovirus	Tomato mosaic virus (strain S-1)	4456887
Tobamovirus	Turnip vein-clearing virus	9629932
Tobamovirus	Youcai mosaic virus	30102450
Tobamovirus	Zucchini green mottle mosaic virus	20889366
Tobravirus	Pea early browning virus	9632339
Tobravirus	Pepper ringspot virus	20178599
Tobravirus	Tobacco rattle virus	20522119
Unassigned	Botrytis virus F	11125723
Unassigned	Oyster mushroom spherical virus	28261418
**Invertebrate**		
Tetraviridae		
Betatetravirus	Nudaurelia capensis beta virus	9631280
Unclassified	Helicoverpa armigera stunt virus	1097407
**Vertebrate**		
Togaviridae		
Alphavirus	Aura virus	21218489
Alphavirus	Barmah forest virus	9629247, 19263412
Alphavirus	Chikungunya virus	22789217
Alphavirus	Eastern equine encephalitis virus	7444405
Alphavirus	Igbo ora virus	9630654
Alphavirus	Mayaro virus	18857923

Alphavirus	Ockelbo virus	334112	■			■	■	■	■	
Alphavirus	O'nyong-nyong virus	3396054	■			■	■	■	■	
Alphavirus	Ross river virus	9790306	■			■	■	■	■	
Alphavirus	Sagiyama virus	7288148	■			■	■	■	■	
Alphavirus	Salmon pancreas disease virus	21321728	■			■	■	■	■	■
Alphavirus	Semliki forest virus	6523491	■			■	■	■	■	
Alphavirus	Sindbis virus	3978526	■			■	■	■	■	
Alphavirus	Sindbis-like virus	3873296	■			■	■	■	■	
Alphavirus	Sleeping disease virus	19352424	■			■	■	■	■	■
Alphavirus	Venezuelan equine encephalitis v.	20800456	■			■	■	■	■	
Alphavirus	Western equine encephalomyelitis v.	21238455	■			■	■	■	■	
Unassigned										
Hepatitis E-like	Hepatitis E virus	34808946	■			■	■	■	■	
Hepatitis E-like	Swine hepatitis E virus	24620203	■			■	■	■	■	
