# Supplementary material for: Bioinformatic mapping of AlkB homology domains in viruses
Source: BMC Genomics. 2005 Jan 3;6:1. doi: 10.1186/1471-2164-6-1 (PMC544882; doi:10.1186/1471-2164-6-1)
Supplement: Additional File 2 — Individual NJ and ML trees for relevant domains (MT, AlkB, HEL, RdRp). [file 1471-2164-6-1-S2.pdf]

Additional file 2

Marit S. Bratlie and Finn Drabløs, “Bioinformatic mapping of AlkB homology domains in viruses”

Phylogenetic trees (Neighbour-Joining and Maximum Likelihood) for MT, AlkB, HEL and RdRp domains from corresponding replicase polyproteins. Please see the main paper for details.

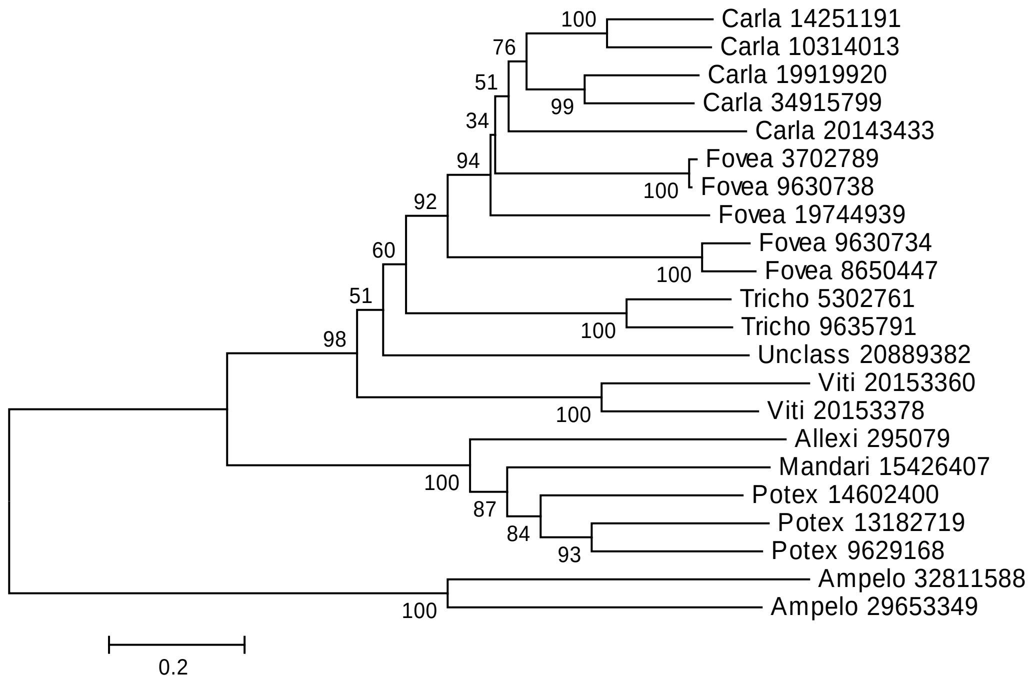

MEGA NJ tree for the MT domain

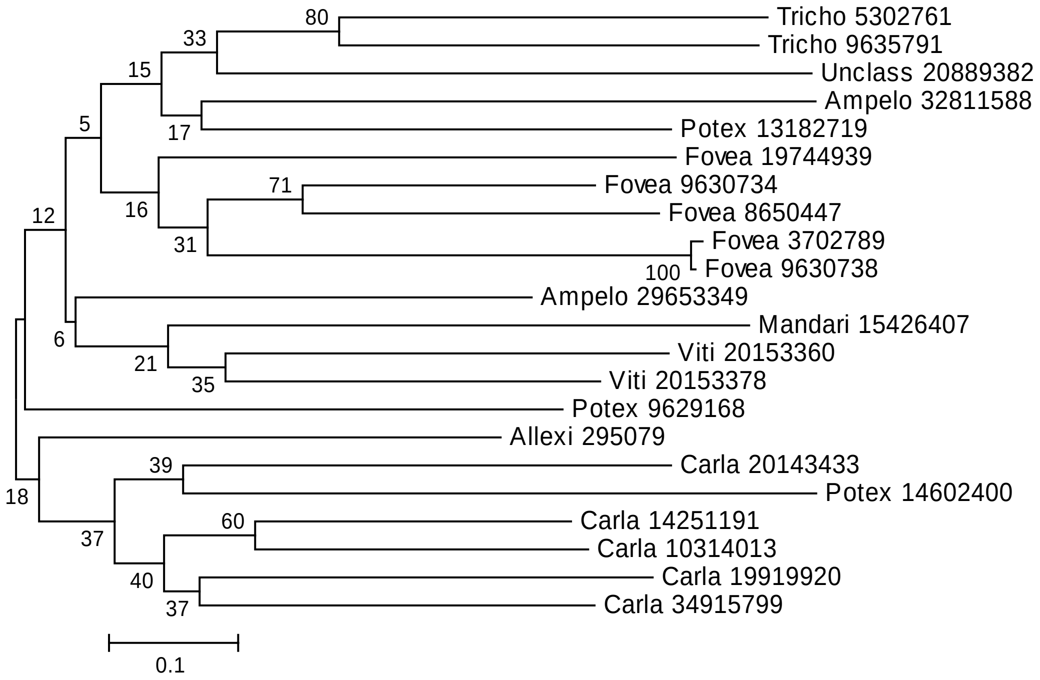

MEGA NJ tree for the AlkB domain

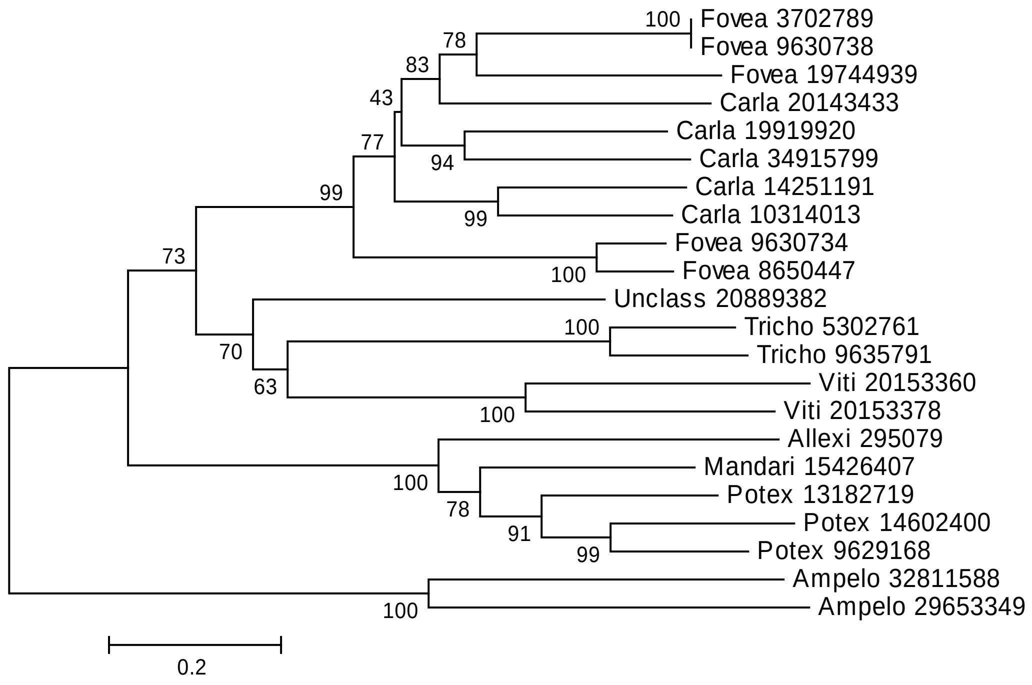

MEGA NJ tree for the HEL domain

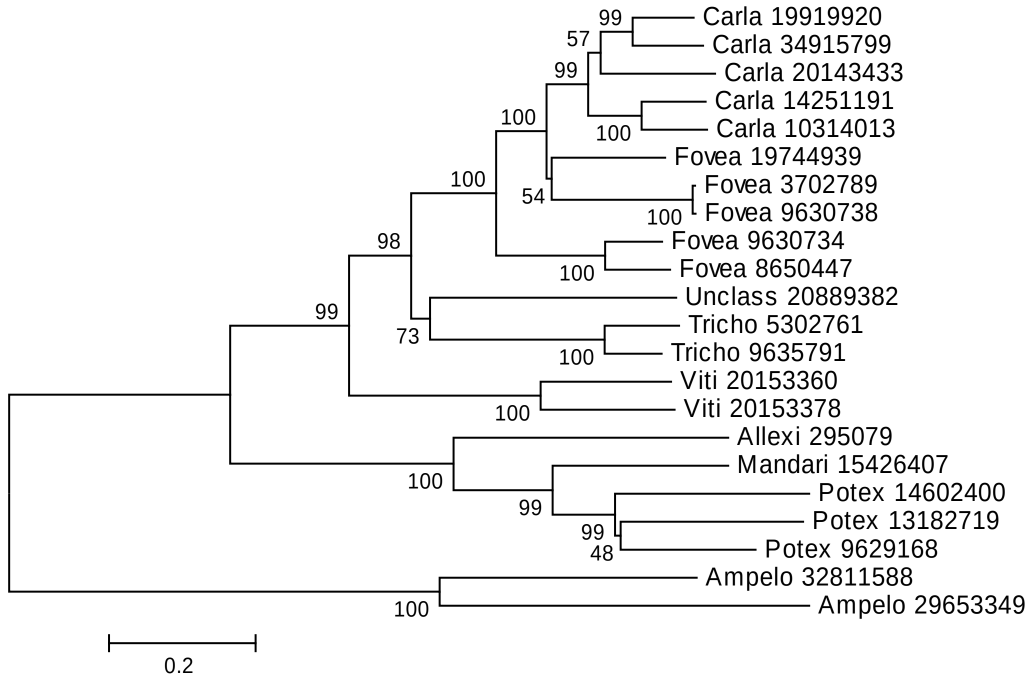

MEGA NJ tree for the RdRp domain

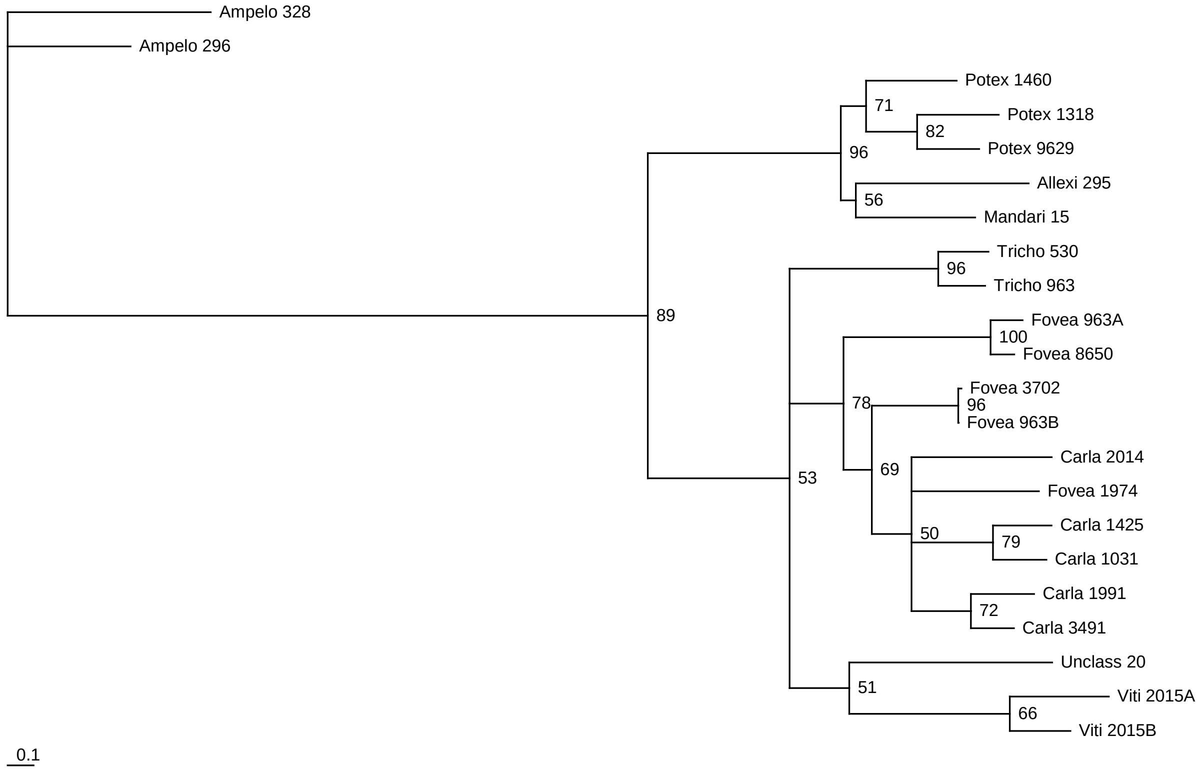

Tree-Puzzle ML tree for the MT domain (drawn with TreeView)

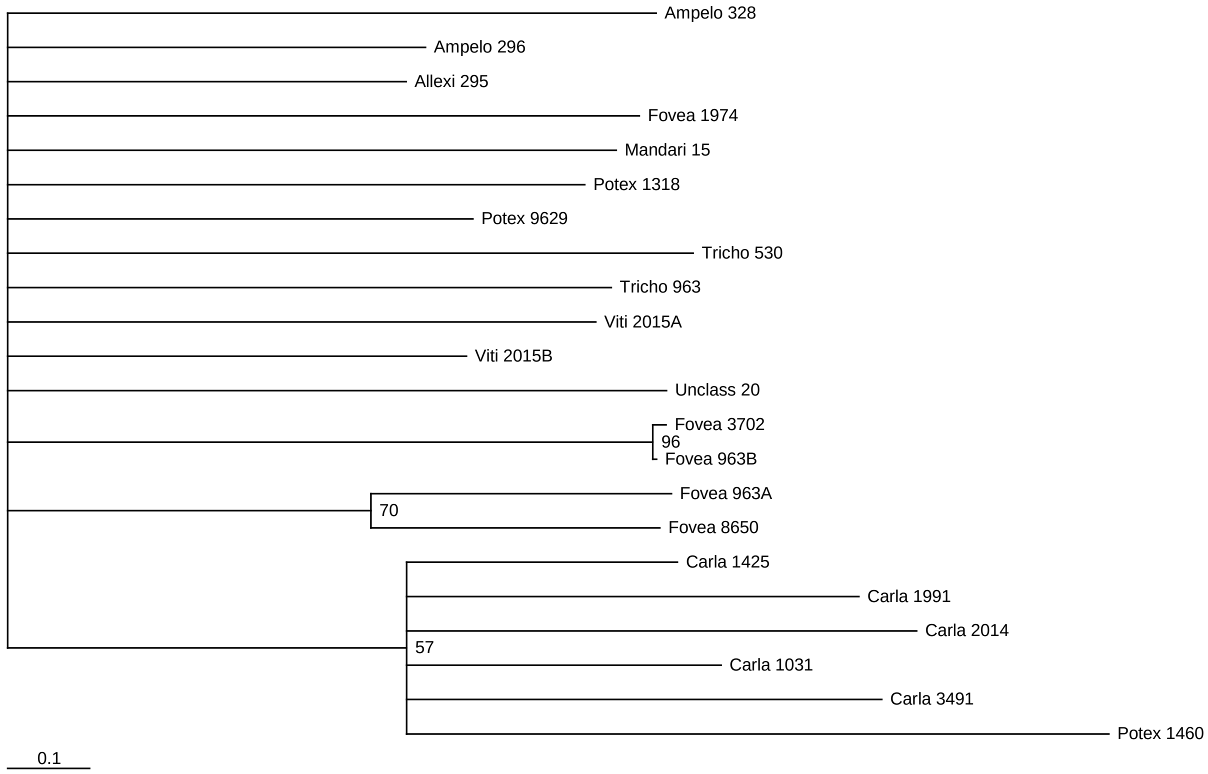

Tree-Puzzle ML tree for the AlkB domain (drawn with TreeView)

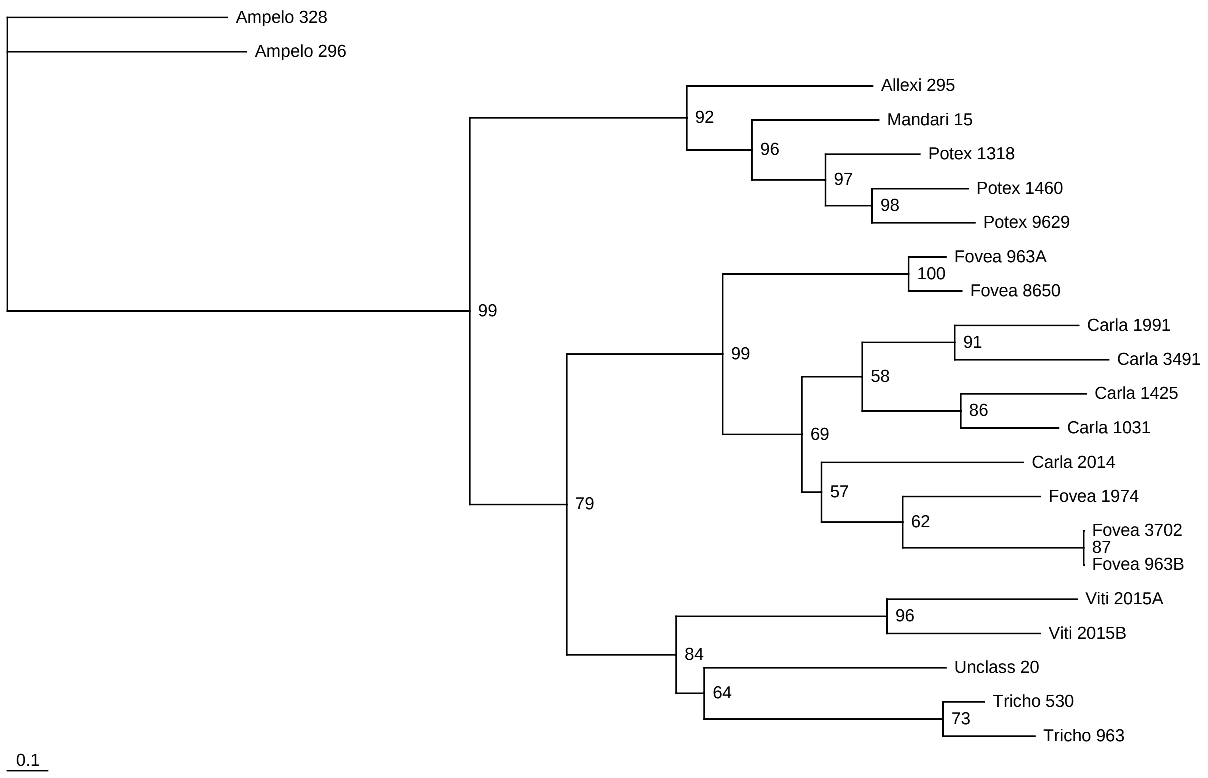

Tree-Puzzle ML tree for the HEL domain (drawn with TreeView)

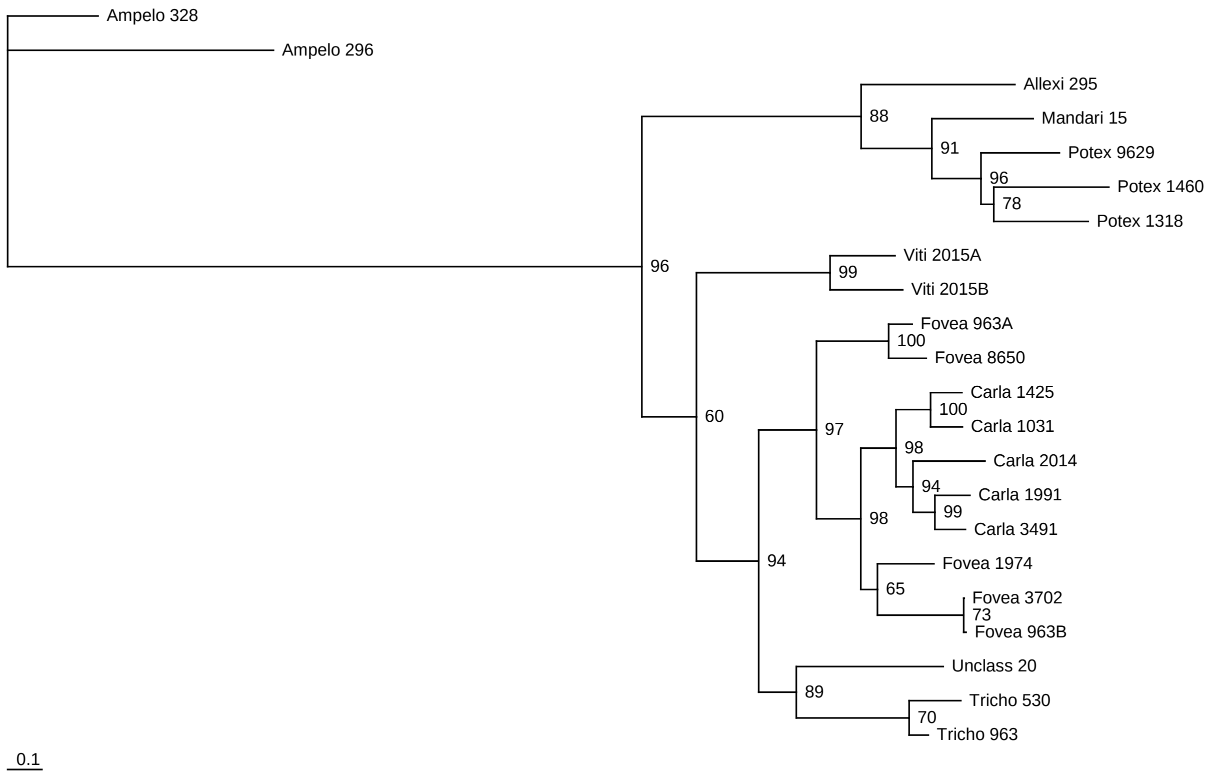

Tree-Puzzle ML tree for the RdRp domain (drawn with TreeView)
